# Supplementary material for: Genes That Associated with Action of ACTH-like Peptides with Neuroprotective Potential in Rat Brain Regions with Different Degrees of Ischemic Damage
Source: Int J Mol Sci. 2025 Jun 28;26(13):6256. doi: 10.3390/ijms26136256 (PMC12249733; doi:10.3390/ijms26136256)
Supplement: Supplementary file 1 [file ijms-26-06256-s001.zip › Supplementary Figure S1.pptx]

## Slide 1
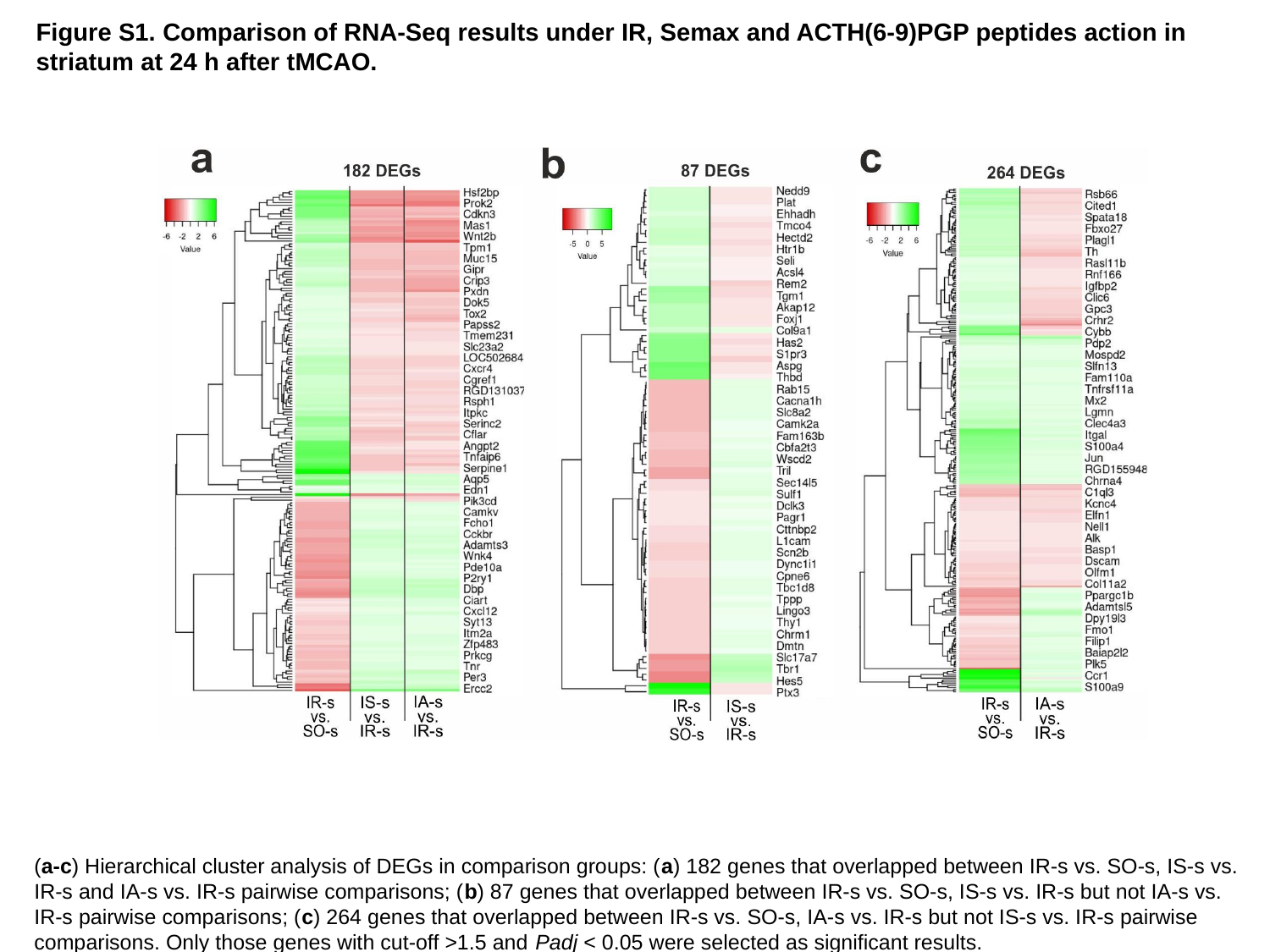

Figure S1. Comparison of RNA-Seq results under IR, Semax and ACTH(6-9)PGP peptides action in striatum at 24 h after tMCAO.
(a-c) Hierarchical cluster analysis of DEGs in comparison groups: (a) 182 genes that overlapped between IR-s vs. SO-s, IS-s vs. IR-s and IA-s vs. IR-s pairwise comparisons; (b) 87 genes that overlapped between IR-s vs. SO-s, IS-s vs. IR-s but not IA-s vs. IR-s pairwise comparisons; (c) 264 genes that overlapped between IR-s vs. SO-s, IA-s vs. IR-s but not IS-s vs. IR-s pairwise comparisons. Only those genes with cut-off >1.5 and Padj < 0.05 were selected as significant results.
