# Supplementary material for: Genes That Associated with Action of ACTH-like Peptides with Neuroprotective Potential in Rat Brain Regions with Different Degrees of Ischemic Damage
Source: Int J Mol Sci. 2025 Jun 28;26(13):6256. doi: 10.3390/ijms26136256 (PMC12249733; doi:10.3390/ijms26136256)
Supplement: Supplementary file 1 [file ijms-26-06256-s001.zip › Supplementary Figure S2.pptx]

## Slide 1
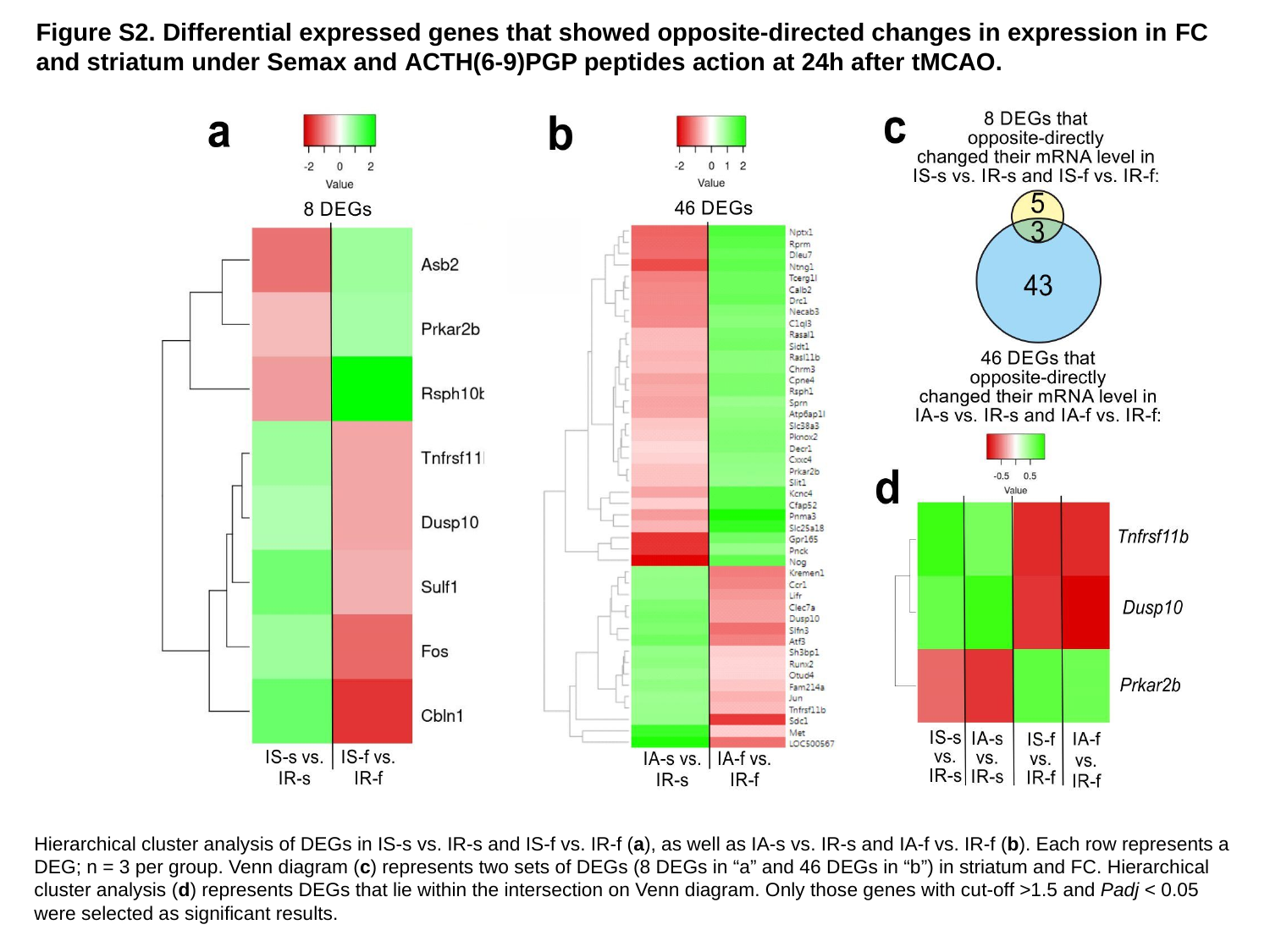

Figure S2. Differential expressed genes that showed opposite-directed changes in expression in FC and striatum under Semax and ACTH(6-9)PGP peptides action at 24h after tMCAO.
Hierarchical cluster analysis of DEGs in IS-s vs. IR-s and IS-f vs. IR-f (a), as well as IA-s vs. IR-s and IA-f vs. IR-f (b). Each row represents a DEG; n = 3 per group. Venn diagram (c) represents two sets of DEGs (8 DEGs in “a” and 46 DEGs in “b”) in striatum and FC. Hierarchical cluster analysis (d) represents DEGs that lie within the intersection on Venn diagram. Only those genes with cut-off >1.5 and Padj < 0.05 were selected as significant results.
