# Supplementary material for: Genes That Associated with Action of ACTH-like Peptides with Neuroprotective Potential in Rat Brain Regions with Different Degrees of Ischemic Damage
Source: Int J Mol Sci. 2025 Jun 28;26(13):6256. doi: 10.3390/ijms26136256 (PMC12249733; doi:10.3390/ijms26136256)
Supplement: Supplementary file 1 [file ijms-26-06256-s001.zip › Supplementary Figure S3.pptx]

## Slide 1
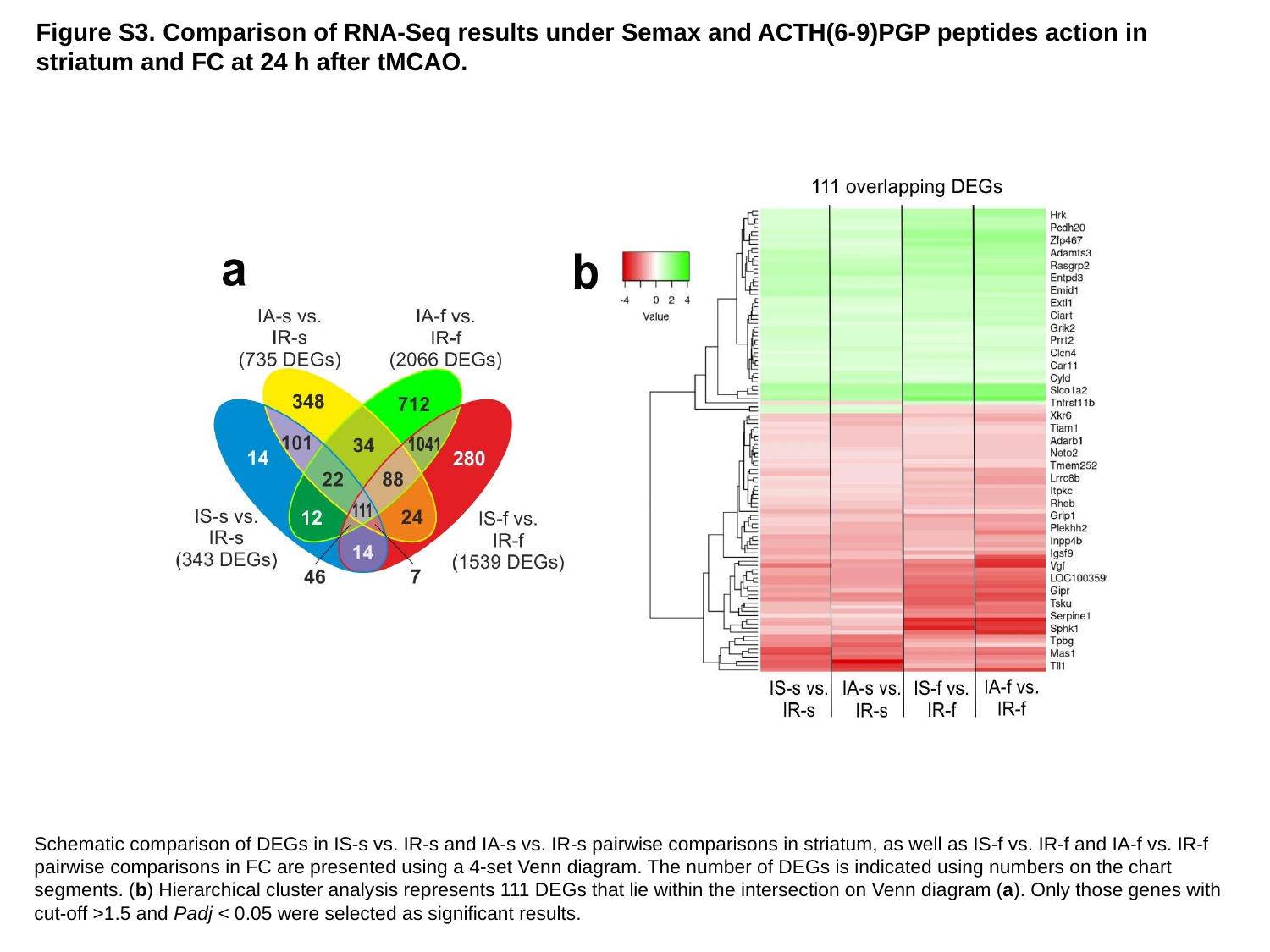

Figure S3. Comparison of RNA-Seq results under Semax and ACTH(6-9)PGP peptides action in striatum and FC at 24 h after tMCAO.
Schematic comparison of DEGs in IS-s vs. IR-s and IA-s vs. IR-s pairwise comparisons in striatum, as well as IS-f vs. IR-f and IA-f vs. IR-f pairwise comparisons in FC are presented using a 4-set Venn diagram. The number of DEGs is indicated using numbers on the chart segments. (b) Hierarchical cluster analysis represents 111 DEGs that lie within the intersection on Venn diagram (a). Only those genes with cut-off >1.5 and Padj < 0.05 were selected as significant results.
