# Supplementary material for: Genes That Associated with Action of ACTH-like Peptides with Neuroprotective Potential in Rat Brain Regions with Different Degrees of Ischemic Damage
Source: Int J Mol Sci. 2025 Jun 28;26(13):6256. doi: 10.3390/ijms26136256 (PMC12249733; doi:10.3390/ijms26136256)
Supplement: Supplementary file 1 [file ijms-26-06256-s001.zip › Supplementary Figure S4.pptx]

## Slide 1
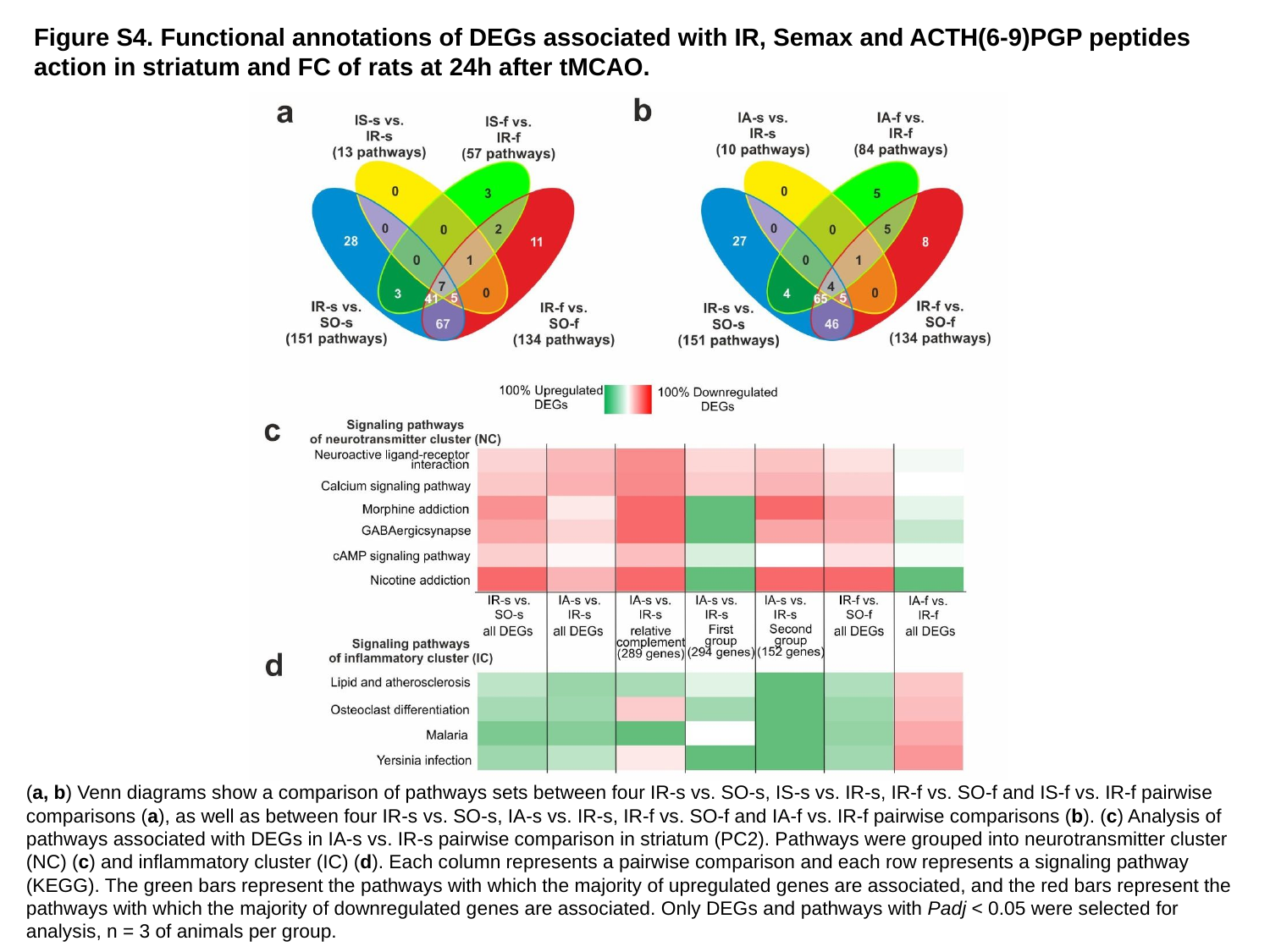

Figure S4. Functional annotations of DEGs associated with IR, Semax and ACTH(6-9)PGP peptides action in striatum and FC of rats at 24h after tMCAO.
(a, b) Venn diagrams show a comparison of pathways sets between four IR-s vs. SO-s, IS-s vs. IR-s, IR-f vs. SO-f and IS-f vs. IR-f pairwise comparisons (a), as well as between four IR-s vs. SO-s, IA-s vs. IR-s, IR-f vs. SO-f and IA-f vs. IR-f pairwise comparisons (b). (c) Analysis of pathways associated with DEGs in IA-s vs. IR-s pairwise comparison in striatum (PC2). Pathways were grouped into neurotransmitter cluster (NC) (c) and inflammatory cluster (IC) (d). Each column represents a pairwise comparison and each row represents a signaling pathway (KEGG). The green bars represent the pathways with which the majority of upregulated genes are associated, and the red bars represent the pathways with which the majority of downregulated genes are associated. Only DEGs and pathways with Padj < 0.05 were selected for analysis, n = 3 of animals per group.
